# Characterization and genome analysis of phage vB_KpnS_SXFY507 against *Klebsiella pneumoniae* and efficacy assessment in *Galleria mellonella* larvae

**DOI:** 10.3389/fmicb.2023.1081715

**Published:** 2023-01-30

**Authors:** Jiao Feng, Fei Li, Li Sun, Lina Dong, Liting Gao, Han Wang, Liyong Yan, Changxin Wu

**Affiliations:** ^1^Institute of Biomedical Sciences, The Key Laboratory of Chemical Biology and Molecular Engineering of Ministry of Education of China, The Key Laboratory of Medical Molecular Cell Biology of Shanxi Province, Shanxi University, Taiyuan, China; ^2^Center for Clinical Laboratory, The Affiliated Taian City Central Hospital of Qingdao University, Taian, China; ^3^College of Life Science and Technology, Beijing University of Chemical Technology, Beijing, China; ^4^Core Laboratory, Shanxi Provincial People’s Hospital (Fifth Hospital) of Shanxi Medical University, Taiyuan, China; ^5^Medical Imaging Center, The Affiliated Taian City Central Hospital of Qingdao University, Taian, China; ^6^Hospital Office, The Affiliated Taian City Central Hospital of Qingdao University, Taian, China

**Keywords:** *Klebsiella pneumoniae*, phage vB_KpnS_SXFY507, *Galleria mellonella*, genome analysis, carbapenem-resistant

## Abstract

Carbapenem-resistant *Klebsiella pneumoniae* is one of the primary bacterial pathogens that pose a significant threat to global public health because of the lack of available therapeutic options. Phage therapy shows promise as a potential alternative to current antimicrobial chemotherapies. In this study, we isolated a new *Siphoviridae* phage vB_KpnS_SXFY507 against KPC-producing *K. pneumoniae* from hospital sewage. It had a short latent period of 20 min and a large burst size of 246 phages/cell. The host range of phage vB_KpnS_SXFY507 was relatively broad. It has a wide range of pH tolerance and high thermal stability. The genome of phage vB_KpnS_SXFY507 was 53,122 bp in length with a G + C content of 49.1%. A total of 81 open-reading frames (ORFs) and no virulence or antibiotic resistance related genes were involved in the phage vB_KpnS_SXFY507 genome. Phage vB_KpnS_SXFY507 showed significant antibacterial activity *in vitro*. The survival rate of *Galleria mellonella* larvae inoculated with *K. pneumoniae* SXFY507 was 20%. The survival rate of *K. pneumonia*-infected *G. mellonella* larvae was increased from 20 to 60% within 72 h upon treatment with phage vB_KpnS_SXFY507. In conclusion, these findings indicate that phage vB_KpnS_SXFY507 has the potential to be used as an antimicrobial agent for the control of *K. pneumoniae*.

## Introduction

*Klebsiella pneumoniae* is a gram-negative bacterium that belongs to the *Enterobacteriaceae* family. It is a part of the healthy microbiome of individuals and colonized in gastrointestinal tract, respiratory tract, urogenital tract, skin, and, nasopharynx (Bengoechea and Sa Pessoa, 2019; Reyes et al., 2019). However, this microorganism attacks immunocompromised individuals and causes diseases, such as pneumonia, liver abscess, urinary tract infection, wound infection, and sepsis (Paczosa and Mecsas, 2016). Carbapenems are widely used to treat infections caused by *K. pneumoniae*, but with the widespread use of such antibiotics, the carbapenem-resistant *K. pneumoniae* (CRKP) appeared (Yigit et al., 2001). Currently, CRKP has disseminated globally and become one of the major bacterial pathogens that pose a significant threat to global public health because of the lack of available therapeutic options (McKenna, 2013; Chen et al., 2014).

Phages are viruses that exclusively infect bacteria with high host specificity, and they are found in all environments where bacteria would generally thrive. Phage therapy is a century-old method that was used for the treatment of bacterial infections. Given the development and spread of MDR *K. pneumoniae*, phage therapy shows promise as a potential alternative to current antimicrobial chemotherapies (Herridge et al., 2020). *Klebsiella* phage can be isolated from a variety of sources, including medical sewage, wastewater, and human intestinal samples (Herridge et al., 2020). However, the current phage database remains limited (Parmar et al., 2017). The isolation and investigation of new phages can enrich the phage database and provide biological options for the treatment of infections caused by multidrug-resistant bacteria.

The *Galleria mellonella* larvae model has been widely used to assess the efficacy of phage therapy (Tsai et al., 2016). Treatment with phage KP1801 significantly reduced the level of *K. pneumoniae* in larvae and improved the survival of *G. mellonella* (Wintachai et al., 2020). Phage BUCT541 significantly increased the survival rate of *K. pneumoniae*-infected *G. mellonella* larvae (Pu et al., 2022b). A similar result was acquired from the assessment of the efficacy of phage BUCT610 against *K. pneumoniae* K1119 in the *G. mellonella* larvae model (Pu et al., 2022a).

In this study, we isolated phage vB_KpnS_SXFY507 against *K. pneumoniae* from hospital sewage. The physiological characteristics of phage vB_KpnS_SXFY507 were investigated, and the genome analysis was performed. The efficacy of phage vB_KpnS_SXFY507 against strain SXFY507 was assessed *in vitro* and the *G. mellonella* larvae model.

## Materials and methods

### Bacterial strain and identification

Carbapenem-resistant *K. pneumoniae* SXFY507 was isolated from a patient in a public hospital in Taiyuan City, China. The strain was incubated in Luria–Bertani (LB) broth at 37°C with shaking at 200 rpm and stored at −80°C in 30% glycerol (v/v).

Bacterial species identification was performed by 16S rRNA gene sequencing (Frank et al., 2008) and by PCR detection of the *K. pneumoniae*-specific *khe* gene (Yin-Ching et al., 2002). The modified CarbaNP test was used to determine the activity of Ambler class A/B/D carbapenemases in bacterial cell extracts (Feng et al., 2017). The major plasmid-borne extended-spectrum beta-lactamase and carbapenemase genes were screened by PCR and Sanger sequencing. The PCR primers used for PCR amplicons were described in previous reports (Chen et al., 2015).

### Phage isolation and purification

The untreated sewage sample was collected from a public hospital in Taiyuan, China. Phage isolation and purification were performed as previously described (Feng et al., 2021; Li F. et al., 2022). Briefly, the sewage sample was centrifuged, and then the supernatant was filtered through a sterile 0.22 μm filter (Millipore, PES membrane). Next, the filtered lysate was co-cultured with *K. pneumoniae* SXFY507 in BHI broth and incubated at 37°C with shaking at 160 rpm for 5 h. After centrifugation, the supernatant was filtered by a sterile 0.22 μm filter. A tenfold dilution series (10^−1^–10^−8^) of the filtered lysate was prepared in SM buffer and then mixed with *K. pneumoniae* SXFY507 followed by incubating at room temperature for 5 min. The mixture was added to 5 ml of top agar (BHI with 0.75% agar), and then poured onto a plate (1.5% agar), and incubated overnight at 37°C to form phage plaques. For phage purification, a single plaque was picked and then resuspended in the SM buffer. Double-layer agar method was performed for forming and screening the phage plaques. The experiment of phage purification was repeated three more times to obtain the purified phage. The purified phage preparation was stored at 4°C for further studies.

### Transmission electron microscopy

The purified phage (4.3 × 10^16^ PFU/ml) was spotted onto a carbon-coated copper grid and then negatively stained with 2% phosphotungstic acid for 5 min. The morphology of phage vB_KpnS_SXFY507 was examined by transmission electron microscopy (JEM-1200EX, JEOL, Japan) at an acceleration voltage of 80 kV.

### Optimal multiplicity of infection determination

The multiplicity of infection (MOI) determination was carried out as previously described (Li F. et al., 2022). Phage vB_KpnS_SXFY507 was incubated with the log-phase culture of *K. pneumoniae* SXFY507 at various proportions (10, 1, 0.1, 0.01, and 0.001) followed by incubation at 37°C 160 rpm for 5 h. The mixture was centrifuged to remove bacterial cells, and the supernatant was filtered by a 0.22 μm filter. The titer of the phage was determined by the double-layer agar method. The proportion that generated the highest phage titer was considered as the optimal MOI.

### One-step growth curve

The one-step growth curve was determined to measure the incubation period and the burst size of the phage (Li F. et al., 2022). Phage vB_KpnS_SXFY507 was incubated with *K. pneumoniae* SXFY507 at an MOI of 0.001. After adsorption at room temperature for 5 min, the mixture was centrifuged at 5,000 × *g* for 10 min at 4°C to remove the unabsorbed phage in the supernatant. The centrifuged precipitation was then resuspended in BHI broth followed by incubation at 37°C 160 rpm. Samples were taken at 10 min intervals within 120 min and then centrifuged the samples to obtain the supernatant. The supernatant was filtered through a 0.22 μm filter and then titrated by the double-layer agar method. The experiment was repeated three times.

### Stability assessment of phage vB_KpnS_SXFY507

To investigate the effect of different thermals and pH on the activity of phage vB_KpnS_SXFY507, the phage was treated with different temperatures and pH values. Briefly, phage vB_KpnS_SXFY507 was incubated at different temperatures (40, 50, 60, 70, and 80°C) and pH values (2.0–12.0) for 60 min, respectively. Then the titers of the treated phage were determined *via* the double-layer agar method.

### Host range analysis

The host range of phage vB_KpnS_SXFY507 was determined by a spot test using 27 *K. pneumoniae* strains (25 clinical strains, ATCC BAA-2146, and, ATCC BAA-1705; Li F. et al., 2022). Briefly, 100 μl of log-phase cultures of each strain was mixed with 5 ml of top agar (BHI with 0.75% agar), and then poured onto individual plates containing solid medium to form a double-layered plate. The plate was incubated at room temperature for a few minutes. Subsequently, 10 μl of phage vB_KpnS_SXFY507 (10^8^ PFU/`) was spotted onto the double-layered plate, and then quiescence the plate at room temperature until the phage suspension was absorbed. The plates were incubated overnight at 37°C to allow the formation of plaques.

### Phage genomic DNA extraction, sequencing, and genome analysis

The genomic DNA of phage vB_KpnS_SXFY507 was extracted using the protease K/SDS method as previously described (Li F. et al., 2022). The NEBNext Ultra II FS DNA Library Prep Kit (NEB) for Illumina was used for library preparation. The genome sequencing of phage vB_KpnS_SXFY507 was performed using the Illumina Novaseq sequencer. Trimmomatic V0.36 program was used to remove adapter regions and the low-quality reads, while using SPAdes v3.13.0 to assemble the complete genomic sequence of phage vB_KpnS_SXFY507.

Open-reading frames (ORFs) were predicted using RAST (Rapid Annotation using Subsystem Technology) 2.0 (Overbeek et al., 2014) combined with BLASTP/BLASTN searches against the UniProtKB/Swiss-Prot (Accessed on 8 July 2022; Consortium, 2021) and RefSeq (https://www.ncbi.nlm.nih.gov/refseq/, Accessed on 8 July 2022) databases. The putative transfer RNA (tRNA)-encoding genes were searched using tRNA scan-SE (Lowe and Chan, 2016). The predicted virulence genes were searched against the virulence factors database (VFDB; Liu et al., 2022), and the antibiotic resistance was analyzed in the comprehensive antibiotic resistance database (CRAD; Alcock et al., 2020) and ResFinder database (Zankari et al., 2012; Bortolaia et al., 2020). Multiple and pairwise sequence comparisons were performed using MUSCLE 3.8.31 (Edgar, 2004) and BLASTN, respectively. Gene organization diagrams were drawn in Inkscape 1.1.

### Phylogenetic analysis

The amino acid sequences of the terminase large subunit (TerL) of indicative phages were aligned using MUSCLE 3.8.31 (Edgar, 2004). Unrooted neighbor-joining trees were generated from the aligned TerL sequences using MEGA7 (Kumar et al., 2016), and evolutionary distances were estimated using the neighbor-joining method with a boot-strap iteration of 1,000.

### Assessment of the efficacy of vB_KpnS_SXFY507 against *Klebsiella pneumoniae* SXFY507 *in vitro*

Phage vB_KpnS_SXFY507 was mixed with the log-phase (OD_600_ = 0.6) *K. pneumoniae* SXFY507 at MOI of 1 and 0.001, respectively. The mixture was incubated at 37°C 160 rpm for 5 h. The OD_600_ and bacterial CFU of the mixture were detected every 1 h within 5 h. The experiment was repeated three times.

### Therapeutic effect of phage vB_KpnS_SXFY507 in the *Galleria mellonella* larvae

*Galleria mellonella* larvae model was used to assess the potential *in vivo* efficacy of phage against *K. pneumoniae* (Thiry et al., 2019). A total of 210 larvae weighting 250–350 mg were selected and divided into seven groups of 10 larvae with technical triplicates. 10 μl of *K. pneumoniae* SXFY507 (1 × 10^5^ CFU/ml) was injected into the last right proleg of larvae by a microsample syringe, and an hour after infection, 10 μl of phage vB_KpnS_SXFY507 was injected into the last left proleg of larvae for treatment at MOI of 100, 10, 1, and 0.001. The other three groups were injected with PBS (10 μl/each), phage vB_KpnS_SXFY507 (10 μl/each, 1 × 10^7^ PFU/ml), and *K. pneumoniae* SXFY507 (10 μl/each, 1 × 10^5^ CFU/ml) on the right last proleg of larvae, respectively. The number of surviving *G. mellonella* larvae was observed and recorded every 8 h for 72 h. The survival curves were plotted using the Kaplan–Meier method, and differences in survival rates between groups were calculated using the log-rank test. Statistical analysis was performed with SPSS statistics software (version 27.0).

### Nucleotide sequence accession number

The complete sequence of *Klebsiella* phage vB_KpnS_SXFY507 genome was submitted to GenBank under accession number ON045001.

## Results

### Molecular characteristics of *Klebsiella pneumoniae* SXFY507

Strain SXFY507 harbored *K. pneumoniae*-specific *khe* gene and demonstrated class A carbapenemase activity. The PCR screening assay of carbapenemase and extended-spectrum beta-lactamases genes detected *bla*_KPC_, *bla*_CTX-M-9G_, and *bla*_SHV_ (but none of the other *bla* genes tested) in *K. pneumoniae* SXFY507.

### Phage isolation and morphology

**Figure 1:**
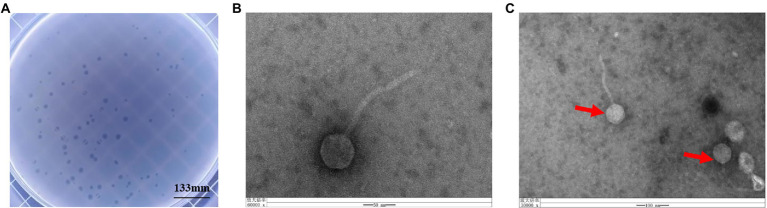
Plaque morphology of bacteriophage vB_KpnS_SXFY507. **(A)** Phage plaques formed of phage vB_KpnS_SXFY507 on the lawn with *Klebsiella pneumoniae* SXFY507. **(B,C)** Transmission electron microscopy of phage vB_KpnS_SXFY507.

Phage vB_KpnS_SXFY507 was successfully isolated from hospital sewage using clinically isolated *K. pneumoniae* SXFY507 as the indicator bacterium. Phage vB_KpnS_SXFY507 formed large, clear plaques on the double-layer plate (Figure 1A). Transmission electron microscopy showed that phage vB_KpnS_SXFY507 exhibited a symmetrical head and a long, non-contractile tail (Figures 1B,C). The head diameter of vB_KpnS_SXFY507 was approximately 55.6 nm, and the tail length was about 174.7 nm. Based on the taxonomic proposal of the International Committee on Taxonomy of Viruses (ICTV), phage vB_KpnS_SXFY507 was considered to belong to the *Siphoviridae* family.

### Physiological characterization of phage vB_KpnS_SXFY507

**Figure 2:**
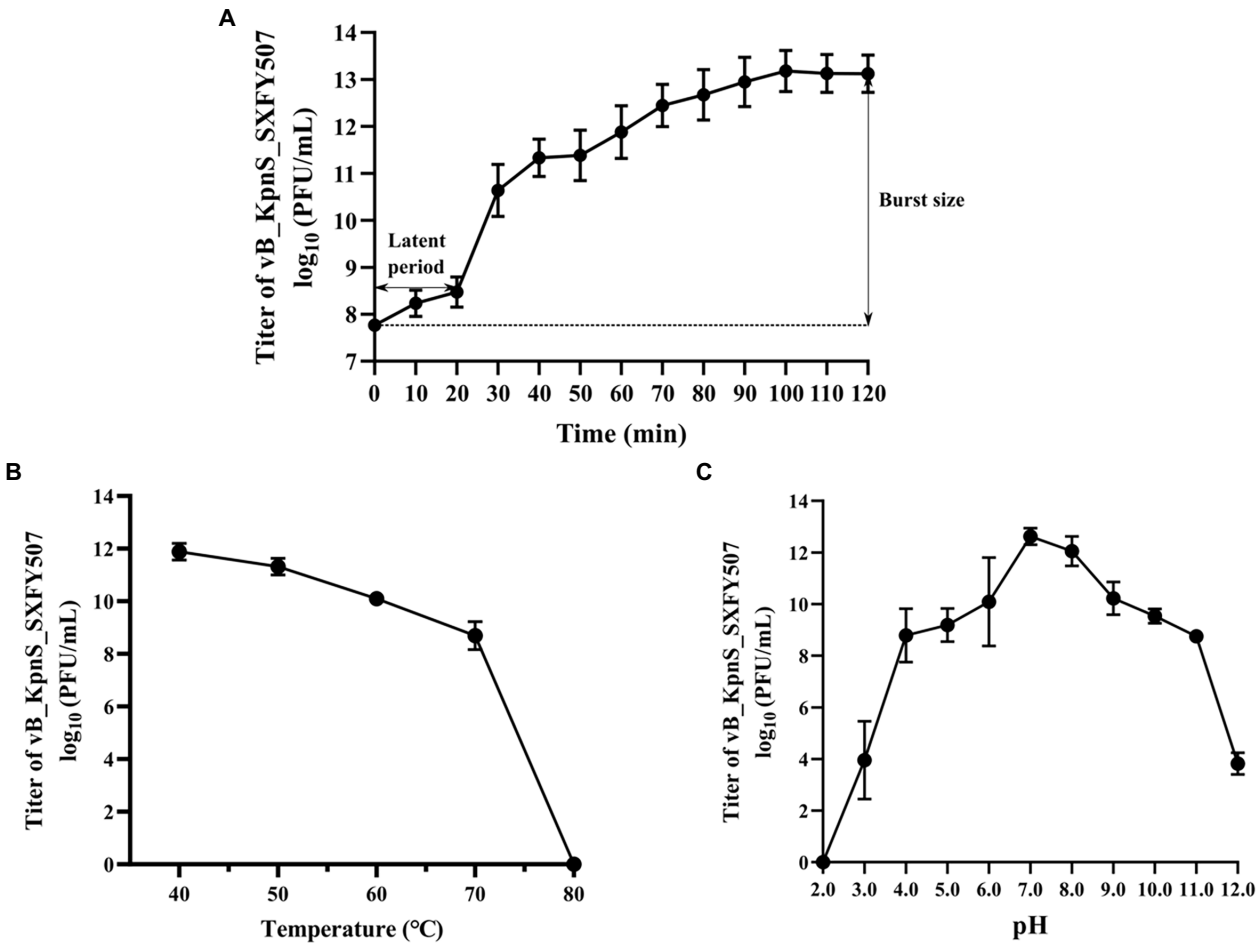
Physiological characterization of phage vB_KpnS_SXFY507. **(A)** One-step growth curve of phage vB_KpnS_SXFY507; **(B)** Thermal stability of phage vB_KpnS_SXFY507; and **(C)** pH stability of phage vB_KpnS_SXFY507. All assays were performed in triplicate.

The titer of phage vB_KpnS_SXFY507 reached maximum value when the MOI was 0.001, indicating that 0.001 was the optimal MOI for the growth of vB_KpnS_SXFY507 (Table 1). The growth parameter of phage vB_KpnS_SXFY507 was determined by one-step growth curve experiment. The latent period of phage vB_KpnS_SXFY507 was approximately 20 min, and the rising phase was about 80 min. The average burst size of this phage was about 246 phages/cell (Figure 2A). Thermal stability tests showed that the titer of phage vB_KpnS_SXFY507 slowly declined from 40 to 70°C, while nearly no phages survived when the temperature reached 80°C (Figure 2B). Phage vB_KpnS_SXFY507 had good lytic activity from pH4.0 to pH11.0. When pH < 4.0 or pH > 11.0, the titer of phage was significantly decreased, and the phage was completely inactivated when pH = 2.0 or pH = 12.0 (Figure 2C). A total of 27 strains of *K. pneumoniae* were collected for host range analysis of phage vB_KpnS_SXFY507. The phage could lyse 23 out of 27 strains, indicating that the lysed range of phages was relatively wide (Supplementary Table S1).

**Table 1:** Optimal MOI assays of phage vB_KpnS_SXFY507.

MOI (PFU/CFU)	Average titer of phage vB_KpnS_SXFY507 (PFU/ml)
10	1 × 10^9^
1	1.63 × 10^11^
0.1	2.14 × 10^11^
0.01	2.3 × 10^11^
0.001	4.7 × 10^11^

### General genomic features of the genome of phage vB_KpnS_SXFY507

**Figure 3:**
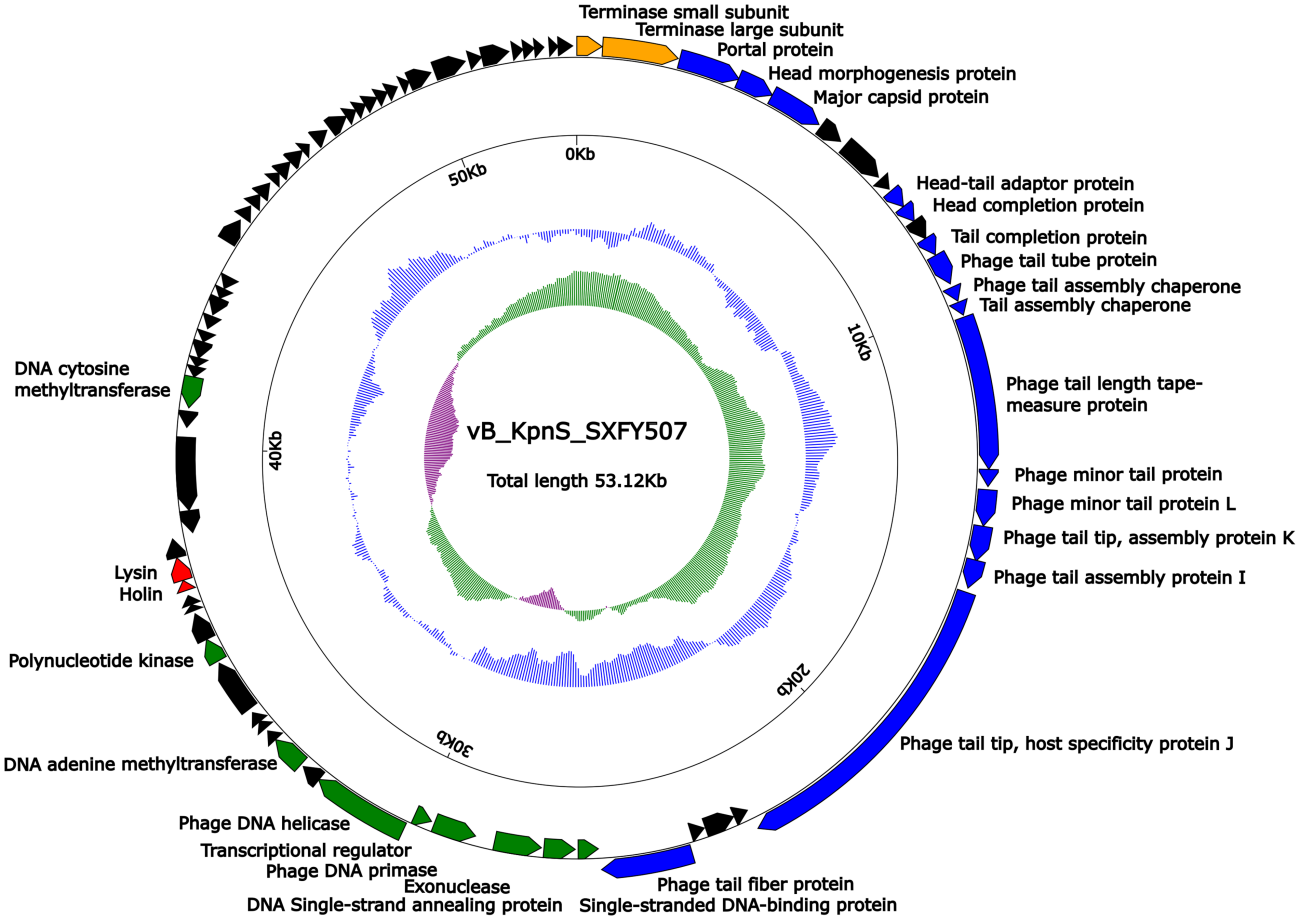
Schematic maps of phage vB_KpnS_SXFY507 genome. Genes are denoted by arrows and colored based on function classification (blue: morphogenesis; green: replication and regulation; orange: DNA packing protein; red: lysis; and black: hypothetical protein). The innermost circle presents GC-skew [(G − C)/(G + C)], with a window size of 500 bp and a step size of 20 bp. The next-to-innermost circle presents GC content.

Genome sequencing showed that the genome of phage vB_KpnS_SXFY507 was a 53, 122-bp circular double-stranded DNA molecule with an average G + C content of 49.1%. According to RAST and BLASTp analysis results, a total of 81 ORFs were predicted in the phage vB_KpnS_SXFY507 genome. Based on the function of ORF, these ORFs in the genome of phage vB_KpnS_SXFY507 were divided into five groups: phage replication and regulation (nine ORFs), morphogenesis (16 ORFs), lysis (two ORFs), DNA-packing protein (two ORFs), and, hypothetical protein (52 ORFs; Figure 3; Supplementary Table S2). In addition, no tRNA, virulence, or antimicrobial resistance gene was discovered in the genome of phage vB_KpnS_SXFY507.

### Comparative genomic analysis

**Figure 4:**
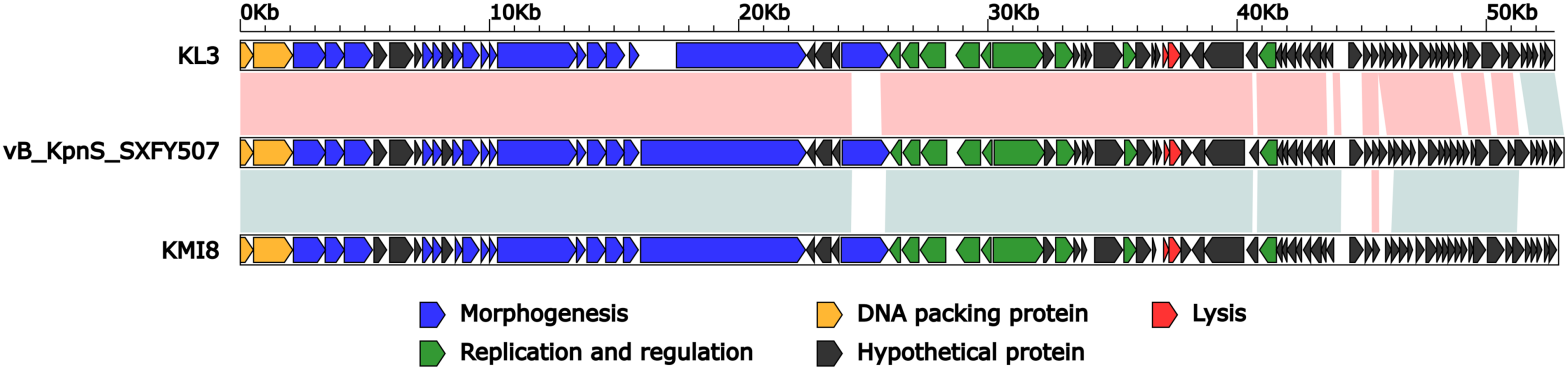
Linear comparison of the genomes of phage vB_KpnS_SXFY507, KL3 (GenBank accession number OK019720), and KMI8 (GenBank accession number MN101222). Genes are denoted by arrows. Genes are colored based on function classification (blue: morphogenesis; green: replication and regulation; orange: DNA packing protein; red: lysis; and black: hypothetical protein). Shading denotes regions of homology (red: >90% nucleotide identity; blue: >85% nucleotide identity).

BLASTN analysis of phage vB_KpnS_SXFY507 genome against the NCBI database showed that vB_KpnS_SXFY507 shared the highest homology (coverage: 93%, identity: 96.9%) with the *Klebsiella* phage KL3 (GenBank accession number OK019720), followed by the *Klebsiella* phage KMI8 (GenBank accession number MN101222) shared 93% coverage and 86.2% identity. The average nucleotide identity (ANI) values between phage vB_KpnS_SXFY507 and phage KL3, phage KMI8 were 96 and 86.33%, respectively. The result indicated that phage vB_KpnS_SXFY507 and phage KL3 but not KMI8 belong to the same group (Goris et al., 2007). Further comparative genomic analysis showed that these three phages displayed two major modular differences: (i) the ORF encoding phage tail fiber protein was different from each other; (ii) some hypothetical proteins were unique in these phages (Figure 4).

**Figure 5:**
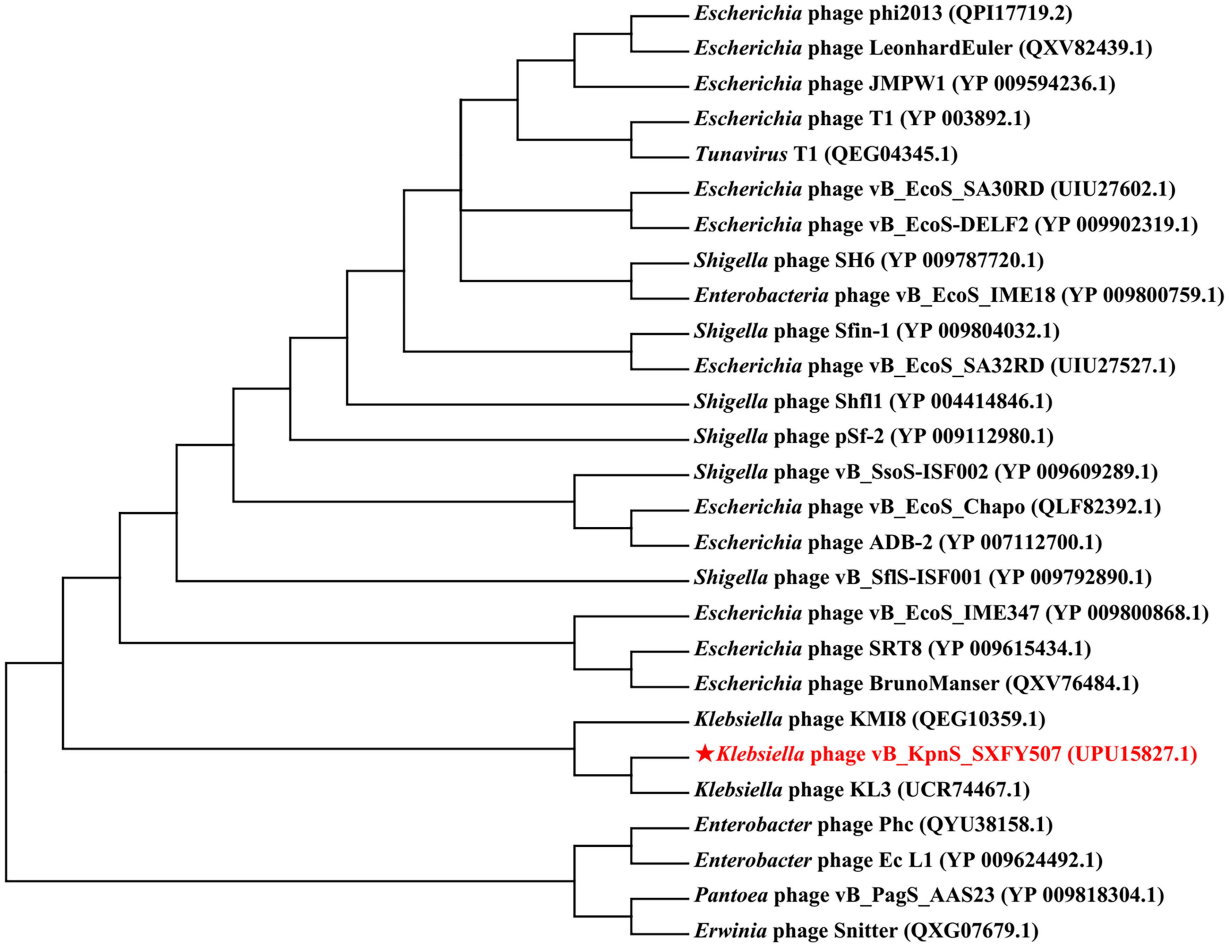
Phylogenetic tree constructed based on the terminase large subunit of phage vB_KpnS_SXFY507. The amino acid sequences of the related phages were downloaded from NCBI.

A phylogenetic tree was constructed based on the TerL amino acid sequence of phage vB_KpnS_SXFY507 and 26 TerL sequences from other phages. The result showed that the TerL of phage vB_KpnS_SXFY507 was closely related to the TerL of *Klebsiella* phage KL3 (Figure 5).

### Assessment of the efficacy of phage vB_KpnS_SXFY507 against SXFY507 *in vitro* and *in vivo*

**Figure 6:**
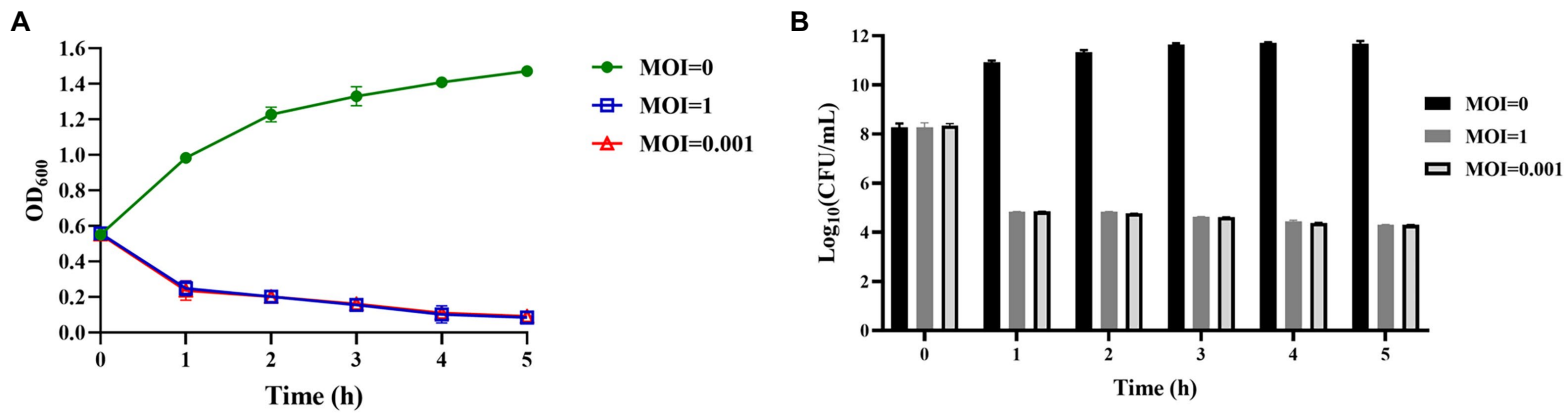
Assessment of the efficacy of phage vB_KpnS_SXFY507 against SXFY507 *in vitro*. **(A)** The variation of OD600 when different titers of phage vB_KpnS_SXFY507 were co-cultured with SXFY507. **(B)** Quantification of bacterial CFU when different titers of phage vB_KpnS_SXFY507 were co-cultured with SXFY507.

**Figure 7:**
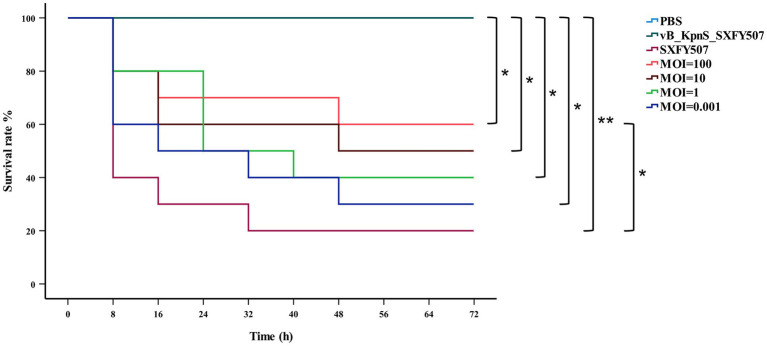
Kaplan–Meier survival curves of *Galleria mellonella* larvae after treatment with phage vB_KpnS_SXFY507 at different MOIs or PBS. Log-rank test was used to determine significance, ^**^p < 0.001, ^*^p < 0.05.

**Figure 8:**
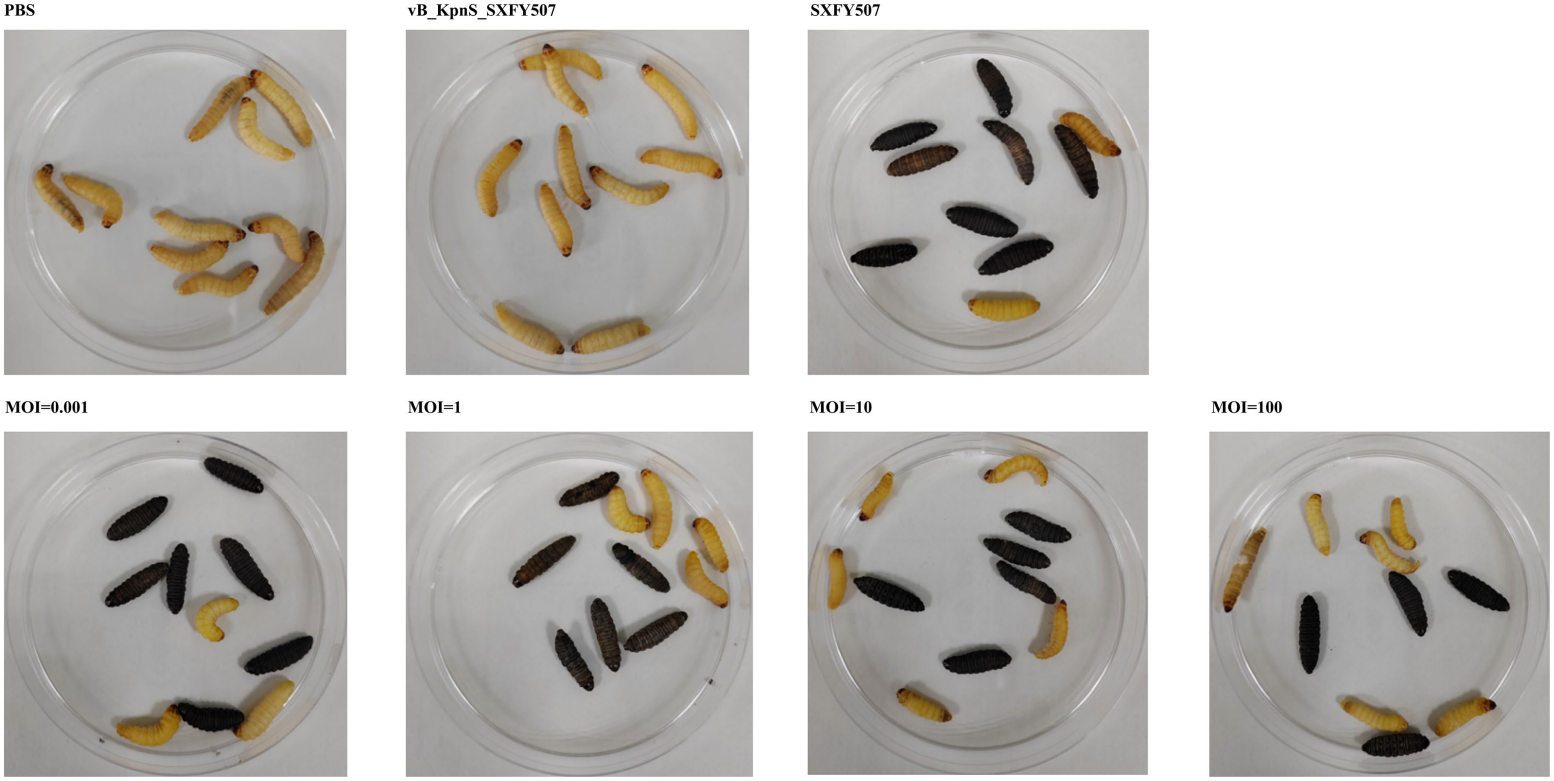
Morphology of *Galleria mellonella* after treatment with phage vB_KpnS_SXFY507.

Phage vB_KpnS_SXFY507 could effectively inhibit the growth of strain SXFY507 *in vitro*. From 0 to 2 h, the OD_600_ and bacterial CFU of the mixture were sharply decreased and maintained at a very low level in 2–5 h compared to the positive control (Figures 6A,B). *Galleria mellonella* larvae model was used to assess the efficacy of phage vB_KpnS_SXFY507 against SXFY507 *in vivo*. We first determined that the optimal inoculum concentration for strain SXFY507 infection was 10^5^ CFU/10 μl, which could induce a mortality rate of 80% in 72 h. In the control group, 20% of larvae inoculated with strain SXFY507 survived. Survival rates of larvae were 60, 50, 40, and 30% when treated with phage at MOI of 100, 10, 1, and 0.001, respectively. Log-rank test revealed that protection of *G. mellonella* larvae by phage at MOI of 100 was significant (value of *p* < 0.05). Both the group of larvae inoculated with phage and the PBS control group were all survived (Figures 7, 8).

## Discussion

Carbapenem-resistant *K. pneumoniae* is one of the significant multidrug-resistant bacterial pathogens that pose a severe threat to global public health (Li et al., 2020). *Klebsiella pneumoniae* SXFY507 has class A carbapenemase activity, and harbored *bla*_KPC_, *bla*_CTX-M-9G_, and *bla*_SHV_. In China, *bla*_KPC_ is the most common carbapenemase resistance gene in the carbapenemase-producing *K. pneumoniae* isolates (Li C. et al., 2022). It is necessary to find alternative strategies for treating infections caused by KPC-producing *K. pneumoniae*. Therefore, it is meaningful to use *K. pneumoniae* SXFY507 as host bacterium to isolate phage and furtherly assess the efficacy of the isolated phage against SXFY507 *in vitro* and *in vivo*.

Phages are highly host-specific, safe, and ubiquitous in the environment (Keen, 2015), so it has been considered as a potential therapeutic approach against highly drug-resistant pathogens, including Carbapenem-resistant *K. pneumoniae* strains (Corbellino et al., 2020). Here, we have isolated a lytic phage vB_KpnS_SXFY507 from hospital sewage using strain SXFY507 as the indicator bacterium. The result suggested that medical sewage may be a better resource for isolating phage against clinical Carbapenem-resistant *K. pneumoniae* strains. For a phage to be useful for phage therapy, it should be better to have a relatively broad host range (Hyman, 2019). Phage vB_KpnS_SXFY507 could lyse 23 out of 27 *K. pneumoniae* strains, indicating that the lysed range of vB_KpnS_SXFY507 was relatively broad.

The physiological characterizations of phages are necessary for their employment. Phage vB_KpnS_SXFY507 had a short latent period of 20 min and a large burst size of 246 PFU/cell. When the MOI was 0.001, the titer of phage vB_KpnS_SXFY507 reached the maximum value. Different pH or temperature conditions may influence the activity of phages, which were intended to be biocontrol agents (Jończyk-Matysiak et al., 2019). Phage vB_KpnS_SXFY507 has a broad range of pH tolerance and good thermal stability. These characteristics make phage vB_KpnS_SXFY507 have the potential to be used as an antimicrobial agent (Jończyk-Matysiak et al., 2019).

Phage vB_KpnS_SXFY507 genome length is 53,122 bp, with G + C content of 49.1%. A total of 81 ORFs were involved in the phage vB_KpnS_SXFY507 genome. Among them, 29 ORFs encoded proteins related to phage replication and regulation, morphogenesis, lysis, and, DNA packing. There were nine ORFs encoded proteins associated with phage replication and regulation. ORF26, ORF28, ORF29, and, ORF31 were predicted as single-stranded DNA-binding protein (SSB), exonuclease, DNA primase, and DNA helicase, respectively. SSBs bind with high affinity to single-stranded DNA, and DNA exonuclease is a multifunctional hydrolase, which plays a role in DNA replication, DNA mismatch repair, and DNA double-strand break repair (Keijzers et al., 2016; Salas et al., 2016). DNA primases catalyze the synthesis of short RNA molecules used as primers for DNA polymerases, and are associated with replicative DNA helicases (Frick and Richardson, 2001).

Phage vB_KpnS_SXFY507 has 16 ORFs encoded proteins that might engage in phage morphogenesis. ORF3 encoded portal protein, which acted as DNA sensors that facilitate packaging and release of the genome (Isidro et al., 2004; Lokareddy et al., 2017; Dedeo et al., 2019). Meanwhile, the proteins encoded by ORF4, ORF5, ORF13, ORF16, and ORF25 were head morphogenesis protein, major capsid protein, tail tube protein, tail length tape-measure protein, and tail fiber protein, respectively.

ORF42 and ORF43 encoded holin and lysin were involved in the lysis module. The DNA-packing proteins in phage vB_KpnS_SXFY507 were terminase small subunit (TerS) encoded by ORF1 and terminase large subunit (TerL) encoded by ORF2. When the TerS recognizes the concatemeric viral DNA, the step of packing phage DNA into procapsids is initiated. Then, the TerL assembles onto the TerS:DNA complex. TerL possesses ATPase and nuclease activity, therefore the TerL can cut the DNA and package it in an ATP-dependent process (Alam and Rao, 2008; Rao and Feiss, 2015).

The remaining 52 ORFs encoded hypothetical proteins, and further study is required. Moreover, no antibiotic resistance or virulence related genes were detected in the genome of phage vB_KpnS_SXFY507, suggesting that phage vB_KpnS_SXFY507 might be theoretically safe for the control of *K. pneumoniae*. Comparative genomic analysis and phylogenetic tree showed that phage vB_KpnS_SXFY507 has a close evolutionary relationship with KL3, and the two phages belong to a new group.

*In vitro*, phage vB_KpnS_SXFY507 showed significant antibacterial activity at MOI of 1 and 0.001, suggesting that it could be used as a biocontrol agent to control the spread of *K. pneumoniae* SXFY507 *in vitro*. *Galleria mellonella* is a flexible and rapid tool to assess phage efficacy (Thiry et al., 2019). Hence, we used *G. mellonella* larvae model to assess the efficacy of phage vB_KpnS_SXFY507 against SXFY507 *in vivo*. In the *G. mellonella* larvae model, the survival rate of larvae inoculated with *K. pneumoniae* SXFY507 was 20%. Based on the *in vitro* experiment, phage at MOI of 1 and 0.001 was chosen for assessing the efficacy of phage vB_KpnS_SXFY507 against SXFY507 *in vivo*. Survival rates of larvae were 40 and 30% when treated with phage at MOIs of 1 and 0.001, respectively. Next, the higher doses (MOI = 10 and 100) of phage vB_KpnS_SXFY507 were used for the treatment experiments. The survival rates were increased to 50 and 60% upon treatment with phage vB_KpnS_SXFY507 at the MOI of 10 and 100. The result was consistent with the previous study that the higher doses of phage led to higher survival rates of *G. mellonella* larvae (Wintachai et al., 2020), but was different from the efficacy of vB_KpnS_SXFY507 against *K. pneumoniae* SXFY507 *in vitro*. We speculate that the difference may be caused by the immunity system of the larvae. The survival rates of phage only and PBS control groups were 100%, demonstrating the safety of the phages in this model. These data showed that Phage vB_KpnS_SXFY507 could efficiently treat *K. pneumoniae* SXFY507 infection.

In conclusion, a lytic phage, named vB_KpnS_SXFY507, against *K. pneumoniae* was isolated and characterized. The physiological characteristics, bioinformatics analysis results, and *G. mellonella* larvae experiment results indicate that the phage vB_KpnS_SXFY507 can be used for phage therapy and is a promising tool for control of *K. pneumoniae*.

## Data availability statement

The datasets presented in this study can be found in online repositories. The names of the repository/repositories and accession number(s) can be found at: https://www.ncbi.nlm.nih.gov/genbank/, ON045001.

## Ethics statement

Written informed consent was obtained from the individual(s) for the publication of any potentially identifiable images or data included in this article.

## Author contributions

JF, LY, and CW conceived the study. JF, FL, LS, LD, LG, and HW performed the experiment and computational analysis. JF, FL, LY, and CW wrote the article. All authors contributed to the article and approved the submitted version.

## Funding

This work was supported by the National Natural Science Foundation of China (82002207) and Program of Graduate Innovation Research of Shanxi Province (2021Y122).

## Conflict of interest

The authors declare that the research was conducted in the absence of any commercial or financial relationships that could be construed as a potential conflict of interest.

## Publisher’s note

All claims expressed in this article are solely those of the authors and do not necessarily represent those of their affiliated organizations, or those of the publisher, the editors and the reviewers. Any product that may be evaluated in this article, or claim that may be made by its manufacturer, is not guaranteed or endorsed by the publisher.

## References

[ref1] Alam T. I. Rao V. B. (2008). The ATPase domain of the large terminase protein, gp17, from bacteriophage T4 binds DNA: implications to the DNA packaging mechanism. J. Mol. Biol. 376, 1272–1281. doi: 10.1016/j.jmb.2007.12.041, PMID: 18234214

[ref2] Alcock B. P. Raphenya A. R. Lau T. T. Y. Tsang K. K. Bouchard M. Edalatmand A. (2020). CARD 2020: antibiotic resistome surveillance with the comprehensive antibiotic resistance database. Nucleic Acids Res. 48, D517–d525. doi: 10.1093/nar/gkz935, PMID: 31665441 PMC7145624

[ref3] Bengoechea J. A. Sa Pessoa J. (2019). *Klebsiella pneumoniae* infection biology: living to counteract host defences. FEMS Microbiol. Rev. 43, 123–144. doi: 10.1093/femsre/fuy043, PMID: 30452654 PMC6435446

[ref4] Bortolaia V. Kaas R. S. Ruppe E. Roberts M. C. Schwarz S. Cattoir V. (2020). ResFinder 4.0 for predictions of phenotypes from genotypes. J. Antimicrob. Chemother. 75, 3491–3500. doi: 10.1093/jac/dkaa345, PMID: 32780112 PMC7662176

[ref5] Chen Z. Li H. Feng J. Li Y. Chen X. Guo X. (2015). NDM-1 encoded by a pNDM-BJ01-like plasmid p3SP-NDM in clinical Enterobacter aerogenes. Front. Microbiol. 6:294. doi: 10.3389/fmicb.2015.00294, PMID: 25926823 PMC4396501

[ref6] Chen L. Mathema B. Chavda K. D. DeLeo F. R. Bonomo R. A. Kreiswirth B. N. (2014). Carbapenemase-producing *Klebsiella pneumoniae*: molecular and genetic decoding. Trends Microbiol. 22, 686–696. doi: 10.1016/j.tim.2014.09.003, PMID: 25304194 PMC4365952

[ref7] Consortium U. (2021). UniProt: the universal protein knowledgebase in 2021. Nucleic Acids Res. 49, D480–D489. doi: 10.1093/nar/gkaa1100, PMID: 33237286 PMC7778908

[ref8] Corbellino M. Kieffer N. Kutateladze M. Balarjishvili N. Leshkasheli L. Askilashvili L. (2020). Eradication of a multidrug-resistant, Carbapenemase-producing *Klebsiella pneumoniae* isolate following oral and intra-rectal therapy with a custom made, lytic bacteriophage preparation. Clin. Infect. Dis. 70, 1998–2001. doi: 10.1093/cid/ciz782, PMID: 31414123

[ref9] Dedeo C. L. Cingolani G. Teschke C. M. (2019). Portal protein: the orchestrator of capsid assembly for the dsDNA tailed bacteriophages and Herpesviruses. Annu. Rev. Virol. 6, 141–160. doi: 10.1146/annurev-virology-092818-015819, PMID: 31337287 PMC6947915

[ref10] Edgar R. C. (2004). MUSCLE: multiple sequence alignment with high accuracy and high throughput. Nucleic Acids Res. 32, 1792–1797. doi: 10.1093/nar/gkh340, PMID: 15034147 PMC390337

[ref11] Feng J. Gao L. Li L. Zhang Z. Wu C. Li F. (2021). Characterization and genome analysis of novel Klebsiella phage BUCT556A with lytic activity against carbapenemase-producing *Klebsiella pneumoniae*. Virus Res. 303:198506. doi: 10.1016/j.virusres.2021.198506, PMID: 34271040

[ref12] Feng J. Yin Z. Zhao Q. Zhao Y. Zhang D. Jiang X. (2017). Genomic characterization of novel IncFII-type multidrug resistant plasmids p0716-KPC and p12181-KPC from *Klebsiella pneumoniae*. Sci. Rep. 7:5830. doi: 10.1038/s41598-017-06283-z, PMID: 28725038 PMC5517477

[ref13] Frank J. A. Reich C. I. Sharma S. Weisbaum J. S. Wilson B. A. Olsen G. J. (2008). Critical evaluation of two primers commonly used for amplification of bacterial 16S rRNA genes. Appl. Environ. Microbiol. 74, 2461–2470. doi: 10.1128/aem.02272-07, PMID: 18296538 PMC2293150

[ref14] Frick D. N. Richardson C. C. (2001). DNA primases. Annu. Rev. Biochem. 70, 39–80. doi: 10.1146/annurev.biochem.70.1.39 11395402

[ref15] Goris J. Konstantinidis K. T. Klappenbach J. A. Coenye T. Vandamme P. Tiedje J. M. (2007). DNA-DNA hybridization values and their relationship to whole-genome sequence similarities. Int. J. Syst. Evol. Microbiol. 57, 81–91. doi: 10.1099/ijs.0.64483-0 17220447

[ref16] Herridge W. P. Shibu P. O'Shea J. Brook T. C. Hoyles L. (2020). Bacteriophages of Klebsiella spp., their diversity and potential therapeutic uses. J. Med. Microbiol. 69, 176–194. doi: 10.1099/jmm.0.001141, PMID: 31976857 PMC7431098

[ref17] Hyman P. (2019). Phages for phage therapy: isolation, characterization, and host range breadth. Pharmaceuticals (Basel) 12:35. doi: 10.3390/ph12010035, PMID: 30862020 PMC6469166

[ref18] Isidro A. Henriques A. O. Tavares P. (2004). The portal protein plays essential roles at different steps of the SPP1 DNA packaging process. Virology 322, 253–263. doi: 10.1016/j.virol.2004.02.012, PMID: 15110523

[ref19] Jończyk-Matysiak E. Łodej N. Kula D. Owczarek B. Orwat F. Międzybrodzki R. (2019). Factors determining phage stability/activity: challenges in practical phage application. Expert Rev. Anti-Infect. Ther. 17, 583–606. doi: 10.1080/14787210.2019.1646126, PMID: 31322022

[ref20] Keen E. C. (2015). A century of phage research: bacteriophages and the shaping of modern biology. BioEssays 37, 6–9. doi: 10.1002/bies.201400152, PMID: 25521633 PMC4418462

[ref21] Keijzers G. Liu D. Rasmussen L. J. (2016). Exonuclease 1 and its versatile roles in DNA repair. Crit. Rev. Biochem. Mol. Biol. 51, 440–451. doi: 10.1080/10409238.2016.1215407, PMID: 27494243

[ref22] Kumar S. Stecher G. Tamura K. (2016). MEGA7: molecular evolutionary genetics analysis version 7.0 for bigger datasets. Mol. Biol. Evol. 33, 1870–1874. doi: 10.1093/molbev/msw054, PMID: 27004904 PMC8210823

[ref23] Li M. Guo M. Chen L. Zhu C. Xiao Y. Li P. (2020). Isolation and characterization of novel lytic bacteriophages infecting epidemic Carbapenem-resistant *Klebsiella pneumoniae* strains. Front. Microbiol. 11:1554. doi: 10.3389/fmicb.2020.01554, PMID: 32793133 PMC7385232

[ref24] Li C. Jiang X. Yang T. Ju Y. Yin Z. Yue L. (2022). Genomic epidemiology of carbapenemase-producing *Klebsiella pneumoniae* in China. Genomics Proteomics Bioinformatics. doi: 10.1016/j.gpb.2022.02.005, PMID: . [Epub ahead of print]. 35307590 PMC10225488

[ref25] Li F. Li L. Zhang Y. Bai S. Sun L. Guan J. (2022). Isolation and characterization of the novel bacteriophage vB_SmaS_BUCT626 against Stenotrophomonas maltophilia. Virus Genes 58, 458–466. doi: 10.1007/s11262-022-01917-5, PMID: 35633495

[ref26] Liu B. Zheng D. Zhou S. Chen L. Yang J. (2022). VFDB 2022: a general classification scheme for bacterial virulence factors. Nucleic Acids Res. 50, D912–D917. doi: 10.1093/nar/gkab1107, PMID: 34850947 PMC8728188

[ref27] Lokareddy R. K. Sankhala R. S. Roy A. Afonine P. V. Motwani T. Teschke C. M. (2017). Portal protein functions akin to a DNA-sensor that couples genome-packaging to icosahedral capsid maturation. Nat. Commun. 8:14310. doi: 10.1038/ncomms14310, PMID: 28134243 PMC5290284

[ref28] Lowe T. M. Chan P. P. (2016). tRNAscan-SE on-line: integrating search and context for analysis of transfer RNA genes. Nucleic Acids Res. 44, W54–W57. doi: 10.1093/nar/gkw413, PMID: 27174935 PMC4987944

[ref29] McKenna M. (2013). Antibiotic resistance: the last resort. Nature 499, 394–396. doi: 10.1038/499394a 23887414

[ref30] Overbeek R. Olson R. Pusch G. D. Olsen G. J. Davis J. J. Disz T. (2014). The SEED and the rapid annotation of microbial genomes using subsystems technology (RAST). Nucleic Acids Res. 42, D206–D214. doi: 10.1093/nar/gkt1226, PMID: 24293654 PMC3965101

[ref31] Paczosa M. K. Mecsas J. (2016). *Klebsiella pneumoniae*: going on the offense with a strong defense. Microbiol. Mol. Biol. Rev. 80, 629–661. doi: 10.1128/mmbr.00078-15, PMID: 27307579 PMC4981674

[ref32] Parmar K. M. Gaikwad S. L. Dhakephalkar P. K. Kothari R. Singh R. P. (2017). Intriguing interaction of bacteriophage-host association: An understanding in the era of omics. Front. Microbiol. 8:559. doi: 10.3389/fmicb.2017.00559, PMID: 28439260 PMC5383658

[ref33] Pu M. Han P. Zhang G. Liu Y. Li Y. Li F. (2022a). Characterization and comparative genomics analysis of a new bacteriophage BUCT610 against *Klebsiella pneumoniae* and efficacy assessment in *Galleria mellonella* larvae. Int. J. Mol. Sci. 23. doi: 10.3390/ijms23148040, PMID: 35887393 PMC9321532

[ref34] Pu M. Li Y. Han P. Lin W. Geng R. Qu F. (2022b). Genomic characterization of a new phage BUCT541 against *Klebsiella pneumoniae* K1-ST23 and efficacy assessment in mouse and *Galleria mellonella* larvae. Front. Microbiol. 13:950737. doi: 10.3389/fmicb.2022.950737, PMID: 36187954 PMC9523250

[ref35] Rao V. B. Feiss M. (2015). Mechanisms of DNA packaging by large double-stranded DNA viruses. Annu. Rev. Virol. 2, 351–378. doi: 10.1146/annurev-virology-100114-055212, PMID: 26958920 PMC4785836

[ref36] Reyes J. Aguilar A. C. Caicedo A. (2019). Carbapenem-resistant *Klebsiella pneumoniae*: microbiology key points for clinical practice. Int. J. Gen. Med. 12, 437–446. doi: 10.2147/ijgm.S214305, PMID: 31819594 PMC6886555

[ref37] Salas M. Holguera I. Redrejo-Rodríguez M. de Vega M. (2016). DNA-binding proteins essential for protein-primed bacteriophage Φ29 DNA replication. Front. Mol. Biosci. 3:37. doi: 10.3389/fmolb.2016.00037, PMID: 27547754 PMC4974454

[ref38] Thiry D. Passet V. Danis-Wlodarczyk K. Lood C. Wagemans J. De Sordi L. (2019). New bacteriophages against emerging lineages ST23 and ST258 of *Klebsiella pneumoniae* and efficacy assessment in *Galleria mellonella* larvae. Viruses 11:411. doi: 10.3390/v11050411, PMID: 31058805 PMC6563190

[ref39] Tsai C. J. Loh J. M. Proft T. (2016). *Galleria mellonella* infection models for the study of bacterial diseases and for antimicrobial drug testing. Virulence 7, 214–229. doi: 10.1080/21505594.2015.1135289, PMID: 26730990 PMC4871635

[ref40] Wintachai P. Naknaen A. Thammaphet J. Pomwised R. Phaonakrop N. Roytrakul S. (2020). Characterization of extended-spectrum-β-lactamase producing *Klebsiella pneumoniae* phage KP1801 and evaluation of therapeutic efficacy *in vitro* and *in vivo*. Sci. Rep. 10:11803. doi: 10.1038/s41598-020-68702-y, PMID: 32678251 PMC7367294

[ref41] Yigit H. Queenan A. M. Anderson G. J. Domenech-Sanchez A. Biddle J. W. Steward C. D. (2001). Novel carbapenem-hydrolyzing beta-lactamase, KPC-1, from a carbapenem-resistant strain of *Klebsiella pneumoniae*. Antimicrob. Agents Chemother. 45, 1151–1161. doi: 10.1128/aac.45.4.1151-1161.2001, PMID: 11257029 PMC90438

[ref42] Yin-Ching C. Jer-Horng S. Ching-Nan L. Ming-Chung C. (2002). Cloning of a gene encoding a unique haemolysin from *Klebsiella pneumoniae* and its potential use as a species-specific gene probe. Microb. Pathog. 33, 1–6. doi: 10.1006/mpat.2002.0499, PMID: 12127794

[ref43] Zankari E. Hasman H. Cosentino S. Vestergaard M. Rasmussen S. Lund O. (2012). Identification of acquired antimicrobial resistance genes. J. Antimicrob. Chemother. 67, 2640–2644. doi: 10.1093/jac/dks261, PMID: 22782487 PMC3468078

