# Supplementary material for: Characterization and genome analysis of phage vB_KpnS_SXFY507 against Klebsiella pneumoniae and efficacy assessment in Galleria mellonella larvae
Source: Front Microbiol. 2023 Jan 30;14:1081715. doi: 10.3389/fmicb.2023.1081715 (PMC9922705; doi:10.3389/fmicb.2023.1081715)
Supplement: Supplementary file 2 [file Table_2.DOCX]

**Table S2. Predicted ORFs in the genome of vB_KpnS_SXFY507**

| **ORF** | **Start** | **Stop** | **Strand** | **Length** | **Classification** | **Putative Function** |
| --- | --- | --- | --- | --- | --- | --- |
| ORF1 | 1 | 522 | + | 522 | DNA packing protein | Terminase small subunit |
| ORF2 | 532 | 2103 | + | 1572 | DNA packing protein | Terminase large subunit |
| ORF3 | 2134 | 3426 | + | 1293 | Morphogenesis | Portal protein |
| ORF4 | 3410 | 4180 | + | 771 | Morphogenesis | Head morphogenesis protein |
| ORF5 | 4177 | 5310 | + | 1134 | Morphogenesis | Major capsid protein |
| ORF9 | 7323 | 7736 | + | 414 | Morphogenesis | Head-tail adaptor protein |
| ORF10 | 7733 | 8107 | + | 375 | Morphogenesis | Head completion protein |
| ORF12 | 8527 | 8919 | + | 393 | Morphogenesis | Tail completion protein |
| ORF13 | 8931 | 9590 | + | 660 | Morphogenesis | Phage tail tube protein |
| ORF14 | 9667 | 9978 | + | 312 | Morphogenesis | Phage tail assembly chaperone |
| ORF15 | 10002 | 10286 | + | 285 | Morphogenesis | Tail assembly chaperone |
| ORF16 | 10326 | 13511 | + | 3186 | Morphogenesis | Phage tail length tape-measure protein |
| ORF17 | 13512 | 13865 | + | 354 | Morphogenesis | Phage minor tail protein |
| ORF18 | 13930 | 14682 | + | 753 | Morphogenesis | Phage minor tail protein L |
| ORF19 | 14685 | 15419 | + | 735 | Morphogenesis | Phage tail tip, assembly protein K |
| ORF20 | 15397 | 15993 | + | 597 | Morphogenesis | Phage tail assembly protein I |
| ORF21 | 16084 | 22713 | + | 6630 | Morphogenesis | Phage tail tip, host specificity protein J |
| ORF25 | 24154 | 26052 | + | 1899 | Morphogenesis | Phage tail fiber protein |
| ORF26 | 26094 | 26531 | - | 438 | Replication and regulation | Single-stranded DNA-binding protein |
| ORF27 | 26594 | 27271 | - | 678 | Replication and regulation | DNA Single-strand annealing protein |
| ORF28 | 27326 | 28348 | - | 1023 | Replication and regulation | Exonuclease |
| ORF29 | 28773 | 29705 | - | 933 | Replication and regulation | Phage DNA primase |
| ORF30 | 29777 | 30163 | - | 387 | Replication and regulation | Transcriptional regulator |
| ORF31 | 30252 | 32285 | + | 2034 | Replication and regulation | Phage DNA helicase |
| ORF33 | 32758 | 33486 | + | 729 | Replication and regulation | DNA adenine methyltransferase |
| ORF38 | 35489 | 35995 | + | 507 | Replication and regulation | Polynucleotide kinase |
| ORF42 | 37079 | 37294 | + | 216 | Lysis | Holin |
| ORF43 | 37297 | 37779 | + | 483 | Lysis | Lysin |
| ORF48 | 40930 | 41607 | - | 678 | Replication and regulation | DNA cytosine methyltransferase |
